# A Gamified Mobile App That Helps People Develop the Metacognitive Skills to Cope With Stressful Situations and Difficult Emotions: Formative Assessment of the InsightApp

**DOI:** 10.2196/44429

**Published:** 2023-06-16

**Authors:** Victoria Amo, Mike Prentice, Falk Lieder

**Affiliations:** 1 Max Planck Institute for Intelligent Systems Tübingen Germany

**Keywords:** ecological momentary interventions, serious games, mindfulness-based interventions, acceptance and commitment therapy, cognitive behavioral therapy, mobile phone

## Abstract

**Background:**

Ecological momentary interventions open up new and exciting possibilities for delivering mental health interventions and conducting research in real-life environments via smartphones. This makes designing psychotherapeutic ecological momentary interventions a promising step toward cost-effective and scalable digital solutions for improving mental health and understanding the effects and mechanisms of psychotherapy.

**Objective:**

The first objective of this study was to formatively assess and improve the usability and efficacy of a gamified mobile app, the InsightApp, for helping people learn some of the metacognitive skills taught in cognitive behavioral therapy, acceptance and commitment therapy, and mindfulness-based interventions. The app aims to help people constructively cope with stressful situations and difficult emotions in everyday life. The second objective of this study was to test the feasibility of using the InsightApp as a research tool for investigating the efficacy of psychological interventions and their underlying mechanisms.

**Methods:**

We conducted 2 experiments. In experiment 1 (n=65; completion rate: 63/65, 97%), participants (mean age 27, SD 14.9; range 19-55 years; 41/60, 68% female) completed a single session with the InsightApp. The intervention effects on affect, belief endorsement, and propensity for action were measured immediately before and after the intervention. Experiment 2 (n=200; completion rate: 142/200, 71%) assessed the feasibility of conducting a randomized controlled trial using the InsightApp. We randomly assigned participants to an experimental or a control condition, and they interacted with the InsightApp for 2 weeks (mean age 37, SD 12.16; range 20-78 years; 78/142, 55% female). Experiment 2 included all the outcome measures of experiment 1 except for the self-reported propensity to engage in predefined adaptive and maladaptive behaviors. Both experiments included user experience surveys.

**Results:**

In experiment 1, a single session with the app seemed to decrease participants’ emotional struggle, the intensity of their negative emotions, their endorsement of negative beliefs, and their self-reported propensity to engage in maladaptive coping behaviors (*P*<.001 in all cases; average effect size=−0.82). Conversely, participants’ endorsement of adaptive beliefs and their self-reported propensity to act in accordance with their values significantly increased (*P*<.001 in all cases; average effect size=0.48). Experiment 2 replicated the findings of experiment 1 (*P*<.001 in all cases; average effect size=0.55). Moreover, experiment 2 identified a critical obstacle to conducting a randomized controlled trial (ie, asymmetric attrition) and how it might be overcome. User experience surveys suggested that the app’s design is suitable for helping people apply psychotherapeutic techniques to cope with everyday stress and anxiety. User feedback provided valuable information on how to further improve app usability.

**Conclusions:**

In this study, we tested the first prototype of the InsightApp. Our encouraging preliminary results show that it is worthwhile to continue developing the InsightApp and further evaluate it in a randomized controlled trial.

## Introduction

### Background

The global prevalence of anxiety and depression has increased substantially in the last decade, exceeding the capacity of the mental health care infrastructure [1]. The importance of developing prevention programs to help people learn how to cope with mental distress in constructive ways before it renders them dysfunctional is evident. Moreover, there are now many people whose well-being could be substantially improved if there were more effective mobile apps for alleviating subclinical levels of anxiety and emotional distress.

Psychotherapy has proven useful in helping people cope with distress, gain perspectives about their mental world, constructively regulate their emotions, and act in accordance with their values [2-4]. Advances in technology and the widespread use of smartphones have created new opportunities to deliver psychotherapy and prevention programs. The use of mobile health apps for mental health has become increasingly popular in the last few years. This includes the use of mobile apps for psychotherapy targeting clinical patients [5,6] and mental well-being apps directed to the general public, with mindfulness apps being the most common [7]. Therefore, technology has greatly expanded what is possible in terms of delivering therapy and conducting psychological research. Ecological momentary interventions (EMIs), for example, are medical treatments delivered via mobile devices that aim to help people integrate intervention strategies into their everyday lives and real-life settings [8]. Previous research has shown that behaviors learned in artificial surroundings do not always translate to real-life scenarios [9]. EMIs can help solve this issue as they allow people to learn new responses to old triggers by training in the same context in which the maladaptive behavior occurs [10]. Thus, people can practice breaking an old habit (eg, smoking) and replacing it with a more adaptive response (eg, mindful breathing) in the real-life situations that trigger the bad habit (eg, after lunch or when other colleagues go for a cigarette). Furthermore, EMIs allow for the collection of data outside clinical settings and the study of whether and how specific therapeutic strategies affect people as they go on with their daily lives and interact with their everyday environments. Therefore, EMIs have great potential for delivering effective interventions in the area of mental health in general and psychotherapy in particular.

One thing that many cognitive psychotherapy modalities have in common is that they teach metacognitive strategies. *Metacognition* refers to the knowledge and cognitive processes involved in the appraisal, monitoring, or control of one’s own mind [11], including monitoring and regulating one’s own thoughts and emotions. This makes it a critical set of skills for coping with mental distress and emotions such as anxiety, sadness, and frustration. *Metacognitive therapy*, for example, is based on the principle that metacognition is key to understanding how cognition operates and how people understand and experience the world around them [12]. Cognitive behavioral therapy (CBT) [13] is based on a model in which emotions are consequences of people’s thoughts and replacing maladaptive thoughts with adaptive ones can help people cope with emotions (cognitive restructuring, reappraisal, or meta-reasoning). *Acceptance and commitment therapy* (ACT) [14] offers a complementary approach that emphasizes improving one’s relationship with thoughts and emotions rather than trying to change them directly, a process that is supported by the act of noticing thoughts as mental events (cognitive defusion) and accepting and embracing emotions and their accompanying bodily sensations as they pass through (acceptance—emotion regulation). Similarly, *mindfulness-based interventions* (MBIs) help people train attention and the capacity to observe mental phenomena nonreactively (meta-awareness) [15]. In the last decade, substantial research has been conducted on the efficacy of different psychotherapies and how they compare with each other. There has also been much discussion regarding the conceptual and technical differences between psychotherapies belonging to different waves of behavioral therapy [16]. For example, it has been discussed how metacognitive-, acceptance-, and mindfulness-based therapies are similar to and different from CBT [17,18] given that their basic premises concerning the causes of mental disorders differ. Some research has focused on understanding common factors across psychotherapies (eg, therapeutic alliance, empathy and listening skills, and cultural adaptation) [19]. However, little research has been conducted to understand the degree to which different psychotherapies train the same underlying metacognitive skills and whether and how they differ from each other at that level.

This motivated us to design, develop, and test the first prototype of the *InsightApp*, an EMI that integrates techniques from different psychotherapies with game elements to train and study the metacognitive mechanisms underlying mental health, belief change, emotion regulation, and behavior change. The InsightApp asks the individual user about their challenges, values, and goals and then uses this information to support them in applying simple metacognitive strategies to their personal triggers and the specific challenges they face throughout the day. The app prompts people to leverage challenging situations and mental distress as an opportunity to train metacognitive skills that are essential for resilience, adaptive emotion regulation, and value-congruent action. Thus, the app supports users in the process of creating adaptive mental and behavioral responses to contextual triggers as they occur.

The InsightApp also aims to allow psychotherapy researchers to study how different psychotherapies affect people’s metacognitive skills and strategies in the real world and how those improvements affect mental health and behavior. As the app allows the tracking of gradual improvement in metacognitive skills and other outcome variables with practice, we believe that it is a valuable tool for studying the effects of metacognition on people’s mental health and behavior in real-life settings. The first prototype of the app integrates therapeutic strategies from CBT, ACT, and MBIs. Specifically, the app helps the user (1) reflect on the content of their thoughts and how they relate to emotions and actions and identify more adaptive alternative thoughts; (2) recognize thoughts and emotions as mental events instead of equating them with reality; and (3) accept and embrace how emotions feel in the body instead of numbing them, suppressing them, or acting them out. This support serves a dual purpose: it alleviates the user’s momentary distress, and it also trains the capacity for adaptive emotion regulation and acting in accordance with one’s goals and values.

### Objectives

Our intent in designing the InsightApp was 2-fold. The first target was to support people in constructively dealing with emotionally challenging situations in real-life settings. The second target was to develop a flexible scientific tool for conducting studies on the efficacy of psychological interventions and answering questions about the underlying psychological mechanisms. In this study, we tested the feasibility of developing an app that achieves both goals simultaneously and improved the InsightApp based on user feedback and efficacy data.

## Methods

### Intervention

#### Overview

The intervention delivered through the InsightApp integrates strategies from CBT, ACT, and MBIs. The key strategies delivered by the app are the *ABCDE method* for cognitive restructuring from CBT [20], as well as reflection on values from ACT [21] and emotion regulation strategies from ACT and MBIs [22]. The ABCDE method aims to help people differentiate between the components of an emotional episode—the activating event (A), the beliefs and thoughts about the event (B), and the consequences of those beliefs in terms of feelings and behaviors (C). Building on this understanding, it then helps the person dispute maladaptive beliefs (D) and find a new effective approach (E). ACT’s reflection on values aims to help people clarify and get in touch with their values so that they can provide inspiration, motivation, and guidance [21]. ACT strategies for embracing mental distress help people construe thoughts as mental events and improve their relationship with difficult emotions by accepting and embracing them when needed. MBIs promote present-moment awareness, focused attention, and a nonjudgmental attitude toward thoughts, among other things. The intervention is divided into 2 main components: the reflection module and the metacognitive coaches. The metacognitive coaches comprise the meta-reasoning coach, which is based on CBT, and the meta-awareness coach, which is based on ACT and MBIs.

#### Reflection Module

The reflection module integrates components of CBT and ACT. The first part of the reflection module incorporates the A and C components from the ABCDE method for cognitive restructuring. In this part, the app guides participants to reflect on a challenging event that provokes strong negative emotions and on suboptimal ways in which they tend to react to that situation. The second part of the reflection module incorporates an ACT reflection on values, which encourages participants to reflect on which values they would like to enact in the situation and how.

#### Metacognitive Coaches

Our mobile app is designed to help train people in the use of 2 fundamental metacognitive skills via 2 coaches: the meta-reasoning coach and the meta-awareness coach. The meta-reasoning coach trains people’s capacity to reflect on the content of their thoughts and how they relate to emotions and actions. The meta-awareness coach helps people train their capacity to recognize thoughts and emotions as mental events and accept and embrace how emotions feel in the body instead of avoiding them, suppressing them, or acting them out.

**Figure 1:**
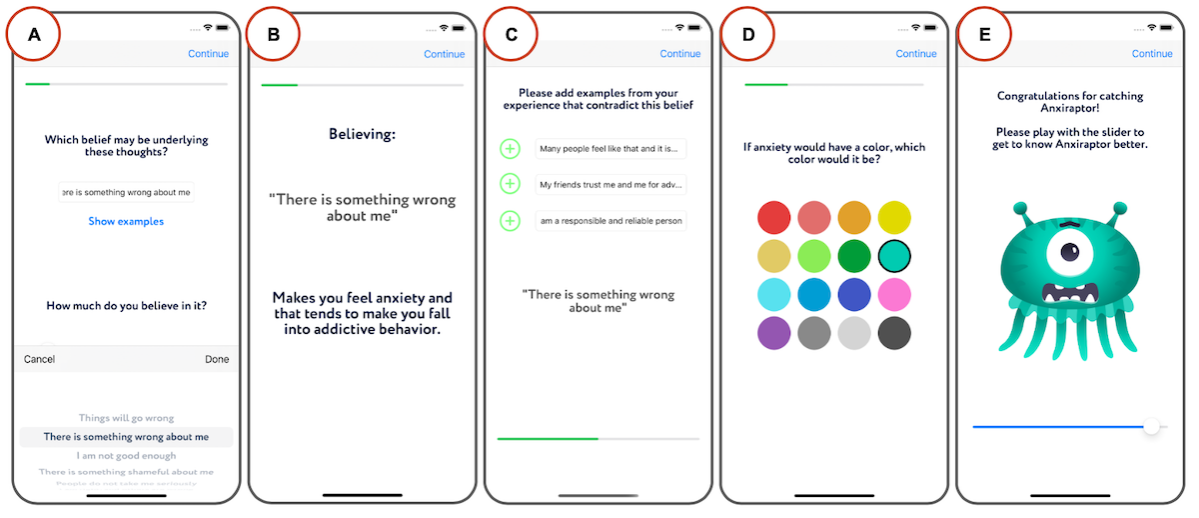
Screenshots from the meta-reasoning coach. The figure shows example screens illustrating how the app guides the user to (A) reflect on the beliefs they hold when they feel a strong negative emotion, (B) reflect on the consequences of holding the maladaptive belief, and (C) challenge the belief. Screenshots D and E exemplify part of the process to customize an avatar representing the recognized maladaptive pattern.

The meta-reasoning coach (Figure 1) integrates steps B, C, D, and E of the ABCDE method for cognitive restructuring (introduced in the *Overview* section). The meta-reasoning coach asks people to reflect on their thoughts and beliefs when they feel a strong negative emotion and when they act in unwanted ways (B). It then asks the user about the consequences of holding those beliefs (C) and guides them to challenge them (D) and consider more empowering alternative beliefs (E). The app helps the user answer the questions of the meta-reasoning coach by providing a list of multiple choices with example answers, as explained in the *Design of the Intervention’s Key Building Blocks* section. In each step, users use sliders to rate the degree of endorsement of thoughts and beliefs. In addition, the app asks users to customize an avatar to represent the recognized maladaptive pattern.

**Figure 2:**
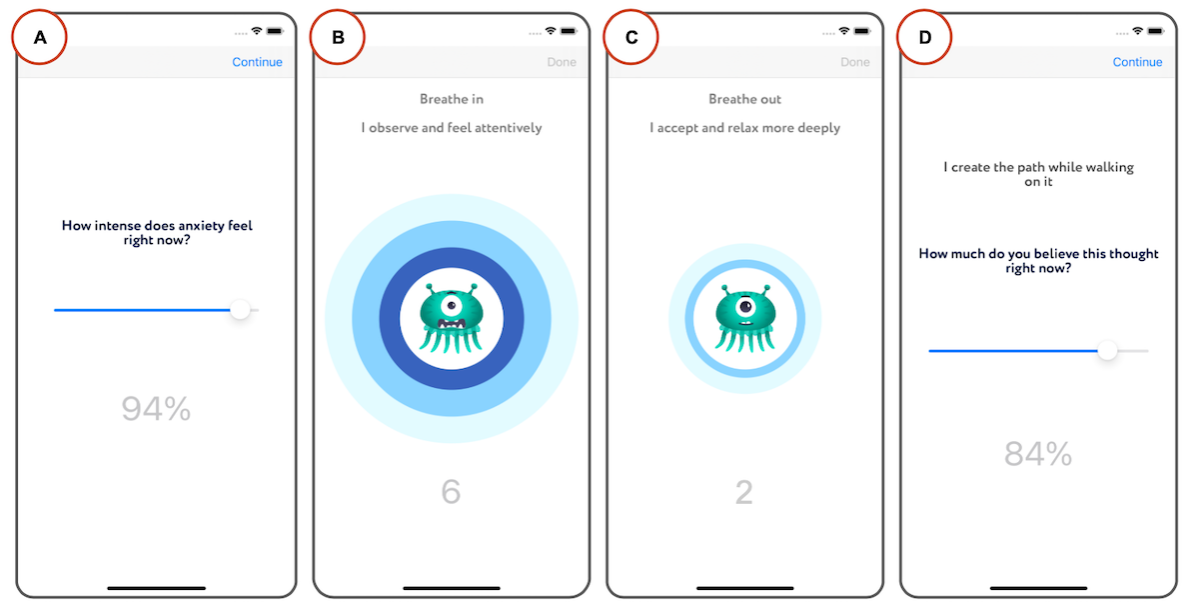
Screenshots from the meta-awareness coach. This figure shows examples of screens that the app uses to guide people to (A) rate the intensity of their emotion before breathing, (B) inhale, (C) exhale, and (D) rate the intensity of their emotion after breathing.

The meta-awareness coach (Figure 2) delivers a breathing meditation to accept and embrace negative emotions. During the meditation, the inhales and exhales are animated with rings that expand and contract. Inhaling is accompanied by the instruction to observe how the emotion feels in terms of bodily sensation, whereas exhaling is accompanied by the instruction to accept those sensations and relax more deeply. As the meditation advances, the previously customized avatar placed in the center of the screen iteratively changes its facial expression from overwhelmed to peaceful and content. Before and after using the meta-awareness coach, the app prompts people to check in on their mental state by rating degrees of affect and beliefs.

The meta-awareness coach is delivered in 2 formats. The first format is a morning practice that is triggered by the device. The second format is the “catch function,” which is available on demand. Users are prompted to complete the morning practice every day at the same time between 5 AM and 11:59 AM. In addition, users are invited to use the “catch function” on demand during the day to catch the little monster avatar each time it is active. Participants are awarded Insight Points every time they complete the morning practice or use the catch function to embrace an emotion. The rationale of Insight Points is to foster a positive view of daily challenges and emotional episodes as valuable opportunities for growth and insight.

### Design of the Intervention’s Key Building Blocks

In this section, we describe the design of the key components of the app, the rationale behind them, and how they can serve as both a therapeutic and scientific tool.

#### Rating Degrees of Affect, Beliefs, and Behavior Using Sliders

**Figure 3:**
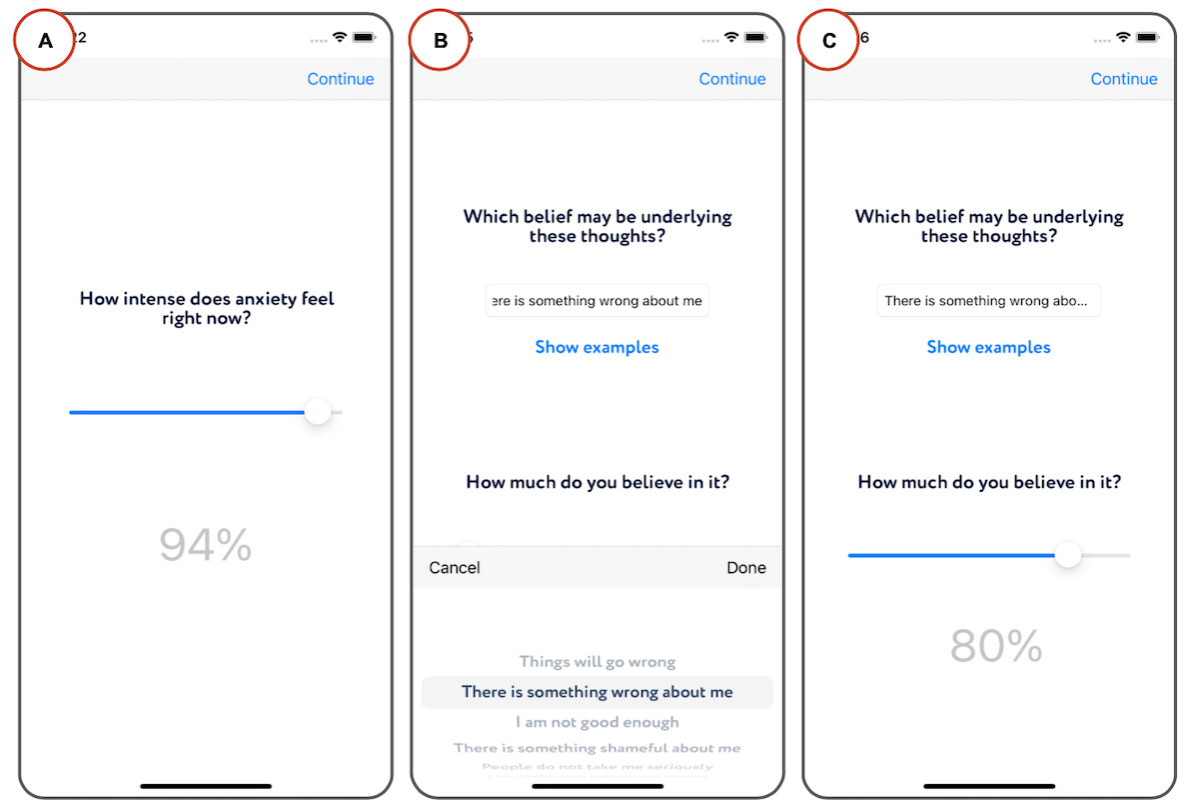
Sliders and questions with example answers. (A) Example screen with a slider for rating the intensity of an emotion. (B) Example screen with a free-text field accompanied by example answers within a picker view. (C) Example of a screen that integrates both components. Sliders are placed directly under a question and are accompanied by a label that shows the current percentage value being selected.

The app asks people to use sliders for rating degrees of affect, beliefs, and behavior on a scale from 0% to 100% (Figure 3A and 3C). The use of sliders for rating has 2 purposes. The first purpose is therapeutic, and the second is scientific. Rating the degree of emotions and thoughts is a technique from cognitive therapy to teach people to distinguish between various degrees in the intensity of their emotions and endorsement of thoughts and beliefs. Among other things, this technique helps people learn emotional awareness and appreciate how emotions and thoughts vary in degree across time and across situations [20]. The sliders for ratings also serve as a scientific tool to collect data regarding a participant’s ongoing mental state. On the basis of self-efficacy research, a scale from 0 to 100 is desirable for sensitive measures (eg, measuring the degree of belief endorsement) as participants tend to avoid extreme values [23]. If the slider scales are appropriate for a particular measure, participants’ responses should be distributed over a large part of the range of alternatives [23]. In experiments 1 and 2, we tested whether this was the case for the measurement of degrees of affect, beliefs, and behavior. In addition, in Multimedia Appendix 1, we present the test-retest reliability of each slider measure.

#### Reflections Accompanied by Example Answers

On multiple occasions, the app asks users to answer questions that require self-reflection. Participants can answer the questions by entering their responses in a text field below the question. Self-reflection questions are accompanied by a list of example answers. The goal of the example answers is to help people better understand the questions and support them in the process of finding answers. Users can access the example answers by clicking on the text field. The app then displays a series of example answers related to the question (Figure 3B). Users are then free to choose one of the example answers or enter their own. To develop the lists of example answers, we conducted a web-based survey in which participants reflected on a strong emotional episode associated with a specific emotion. We asked participants to specify the situation they were going through, their thoughts and beliefs, and the negative ways in which they tended to act. In addition, we asked them to reflect on positive alternative ways in which they could have interpreted the situation and how that interpretation would make them feel and act. To give participants a better understanding of what we were asking, we provided them with example stories that specified an answer to each question. To create the final lists of example answers, we extracted repeated patterns from participants’ answers and converted them to sentences that were not context dependent.

### Ethical Considerations, Informed Consent, and Participation

Experiments 1 and 2 were conducted according to protocol 510/2020BO approved by the Independent Ethics Commission at the Faculty of Medicine of the University of Tübingen.

The *Data Protection and Privacy Policy* protocol was authorized by the Max Planck Society Data Protection Office. The authorization affirms that the project proposal complies with the internal data protection requirements of the Max Planck Society as well as with the General Data Protection Regulation (European Union 2016/679) and the German Federal Data Protection Act. The Data Protection and Privacy Policy agreements are included in the ethics protocol.

To participate in experiments 1 and 2, participants had to consent to (1) take part in the study and (2) share their nonidentifiable data for research purposes. By giving consent, participants agreed to the participation requirements, time commitment, payments, bonuses, privacy policy, data protection, data collection, data use, and withdrawal process.

Participants were recruited and compensated via Prolific (Prolific Academic Ltd). All participants earned a base payment of £6 (US $7.58) per hour. Depending on their participation, they could increase their wages to up to £14.5 (US $18.32) per hour. For more information about Prolific and the exact payment and bonus system, please refer to the *Participants* section of each experiment.

### Experiment 1

In the first experiment, we used a pretest-posttest design to test how effective a single session with the app was at helping people improve their current mental state. This was accomplished by decreasing the intensity and degree of struggle with negative emotions, decreasing the endorsement of maladaptive beliefs, increasing the endorsement of adaptive beliefs, decreasing the perceived likelihood of acting in unwanted ways, and increasing the perceived likelihood of acting in ways that they valued.

#### Participants

We recruited and compensated participants via Prolific, a web-based platform for conducting web-based research. Prolific allows researchers to efficiently recruit and remunerate a large number of participants for web-based studies. This platform is used by >25,000 researchers. Prolific gives researchers access to a pool of 130,000 potential participants. Prolific is available for participants from all countries belonging to the *Organization for Economic Co-operation and Development* except for Turkey, Lithuania, Colombia, and Costa Rica. All participants on Prolific are aged ≥18 years. The platform allows researchers to screen participants depending on their characteristics, such as gender, educational level, and system of beliefs, among many others. Compared with other platforms for web-based studies, Prolific is known for providing superior data quality [24].

The sample consisted of 65 participants (completion rate: 63/65, 97%) from a population of approximately 45,000 international, English-speaking, and iPhone-owning adults on Prolific. Of these 65 participants, 2 (3%) were excluded from the analyses for not completing the intervention, leaving a final sample of 63 participants. Of these 63 participants, 60 (95%) shared demographic information (mean age 27, SD 14.9; range 19-55 years; 41/60, 68% female). The sample included participants who identified as Asian (4/60, 7%), Black (8/60, 13%), White (37/60, 62%), mixed (8/60, 13%), and other (2/60, 3%). Participants’ continents of residence were Europe (36/60, 60%), Africa (11/60, 18%), America (12/60, 20%), and other (1/60, 2%). Each participant received a compensation of £6 (US $7.58) plus a bonus of £2 (US $2.53) for finishing the study. The mean completion time for the experiment was 33 minutes, ranging from 15 to 95 minutes.

#### Outcome Measures

Table 1 summarizes the outcome measures collected by the app for experiment 1. The outcome measures are delivered twice: first as part of the reflection module and meta-reasoning coach and then directly after participants interact with the metacognitive coaches.

**Table 1:** Single-item outcome measures.^a,b^

Outcome variable	Question	Scale
Intensity of the emotion	How intense does [emotion] feel right now?	0%-100%
Strength of the struggle	How strong is the struggle with [emotion] right now?	0%-100%
Likelihood of unwanted action	How likely are you to [unwanted action] in your current emotional state?	0%-100%
Likelihood of valued action	How likely are you to [valued action] in your current emotional state?	0%-100%
Strength of maladaptive belief	How much do you believe this thought right now?	0%-100%
Strength of adaptive belief	How much do you believe this thought right now?	0%-100%

^a^The app asks participants to use a slider (0% to 100%) to rate (1) the intensity of the negative emotion they are feeling, (2) the degree to which they are struggling with the emotion, (3) the current perceived likelihood of acting in unwanted ways, (4) the current perceived likelihood of acting in a value-congruent way, (5) their endorsement of the maladaptive belief, and (6) their endorsement of the adaptive belief.

^b^Brackets designate customized text within the questions that display participants’ previous choices.

#### User Experience Survey

The InsightApp asks participants to rate the app (on a scale from 1 to 5 stars) and answer the following open-ended questions: “What did you like or not like about the App?” “Did you find something confusing?” “Did you find something positively surprising?” and “In which moments would you use the app?” In addition, the app asks participants to use a 6-point Likert scale (strongly disagree=1; strongly agree=6) to specify the degree to which they agree with the following statement: “I developed a valuable skill for my daily life.”

#### Procedure

Participants were first directed to a web-based form where they received instructions on how to set up their phones and download the “InsightAppExperiment” from the App Store. Before starting the experiment, participants provided informed consent to participate in the study and share their data. In the first part of the experiment, participants watched a 1-minute video that explained the onboarding process and entered basic information about their gender, age, and nationality. To continue, participants completed the reflection module*.* Participants then continued to the intervention module, where they watched a 1-minute video with instructions and were guided by the app through the meta-reasoning and meta-awareness coaches, as described in the *Intervention* section. Finally, we asked participants to report on the outcome measures and complete the user experience survey.

#### Statistical Analysis

The distributions of many pre- and posttest scores on outcome measures were highly skewed and violated the normality assumption according to a Shapiro-Wilk test with a significance level of Cronbach α=.05. As such, we used the nonparametric paired-sample Wilcoxon test (PSW) [25,26] to compare participants’ pre- and posttest scores on outcome measures.

We computed descriptive statistics to analyze the numerical results of the user experience survey. To analyze the results for open-ended questions, we extracted common categories by analyzing participants’ answers. We then classified their answers according to those categories and used descriptive statistics. In addition, we summarized the participants’ feedback on each question when provided.

### Experiment 2

#### Overview

Experiment 2 had 2 purposes. The first purpose was to explore how effective the app’s morning practice was when people used the app on a daily basis. The second purpose was to test the feasibility of conducting longitudinal experiments using the app. To test the effectiveness of the InsightApp’s morning practice in a longitudinal study, we asked participants to complete a practice session with the app every morning for at least 5 out of 7 days. We used pretest-posttest scores to calculate how the practice session with the app influenced their mental state on average when measured repeatedly. In parallel, we tested the feasibility of conducting longitudinal experiments using the app by randomly assigning participants to the experimental condition or a control condition. We measured the 2 conditions’ attrition rates to inform the planning and design of a future randomized controlled trial.

#### Participants

The sample consisted of 200 participants (completion rate: 142/200, 71%) from a population of approximately 45,000 international, English-speaking, and iPhone-owning adults on Prolific (for more information about Prolific, please refer to the *Participants* section of experiment 1). Of these 200 participants, 7 (3.5%) were excluded from the study during onboarding for failing attention checks; those participants were replaced with new ones. Of the 200 participants who started the longitudinal study, 142 (71%) finished. A total of 25% (50/200) of the participants were excluded for not completing a predefined minimum number of participation days. In total, 3.5% (7/200) of the participants could not continue because of a technical problem (ie, they changed their phones during the intervention or the app crashed), and 0.5% (1/200) of the participants were excluded from the study for failing ≥2 attention checks during the offboarding. The final sample of 142 participants consisted of 59 (41.5%) from the experimental condition and 83 (58.5%) from the control condition (mean age 37, SD 12.16; range 20-78 years; 78/142, 54.9% female). The sample included participants who identified as Asian (10/142, 7%), Black (6/142, 4.2%), White (119/142, 83.8%), mixed (5/142, 3.5%), or other (2/142, 1.4%). The payment for the study was divided into 2 parts. Participants received the first payment for successfully downloading the app, completing the onboarding process, and starting the experiment. The amount was calculated to achieve an average wage of £6 (US $7.58) per hour. After completing the experiment on day 14, participants were paid for their daily participation, offboarding, and extra bonuses. By actively participating and completing the study, participants could increase their wages up to £12.11 (US $15.30) per hour. All participants received a bonus of £4 (US $5.05) for completing the study. In addition, we included a participation bonus of up to £7 (US $8.85), which was cut in half each time a participant missed a day. Participants who were excluded were paid for their work completed until the point of exclusion, with a base payment rate of £6 (US $7.58) per hour. The estimated average time spent in the study was 60 minutes for the control condition and 80 minutes for the experimental condition.

#### Outcome Measures

##### Attrition Rate

Participants were excluded from the experiment if they failed the attention check or missed their daily goal >4 out of 13 days. Participants failed the attention check if they took <1.6 seconds to answer ≥15 survey questions or if they answered ≥3 reflection questions in <3 seconds. We selected the minimum answer time and the minimum number of answers by inspecting the answer times obtained when testing the app before conducting experiments 1 and 2. The daily goal for participants in the control group was to fill in a daily report every evening. The daily goal for participants in the experimental group was also to fill in a daily report every evening and, in addition, complete the morning practice during 7 days of the intervention.

We calculated the attrition rate as the ratio of the number of participants who dropped out to the total number of participants who started the experiment. We calculated the adherence rate as the ratio of the number of participants who completed the study to the total number of participants who started the experiment. To fulfill the criteria for starting the study, participants had to successfully complete the onboarding section and enter a valid completion code on the Prolific platform.

##### Outcome Measures Administered Before and After the Morning Practice

The morning practice delivered the same outcome measures presented in experiment 1 (Table 1) excluding the perceived likelihood of unwanted and valued action. Outcome measures were delivered directly before and directly after participants interacted with the metacognitive coaches.

##### Outcome Measures Administered in the Evening Report

Table 2 summarizes the outcome measures collected in the evening report. We asked participants to report on their days each day of the study between 7 PM and midnight.

**Table 2:** Single-item outcome measures administered during the evening report.^a,b^

Outcome variable	Question	Scale
Stressor	On a scale from 1 to 10, how severe or problematic was the situation today?	0-10
Enactment of unwanted action	To what extent did you [unwanted action] today?	0%-100%
Enactment of valued action	To what extent did you [valued action] today?	0%-100%
Intensity of the emotion	How intense did [emotion] feel today?	0%-100%
Strength of the struggle	How strong did the struggle with [emotion] feel today?	0%-100%

^a^In the evening report, participants first rated how stressed they felt by the predefined situation that day on a scale from 0 to 10. They then specified the following with a slider (0% to 100%): (1) to what extent they enacted the predefined unwanted action, (2) to what extent they enacted the predefined valued action, (3) the intensity of the emotion, and (4) the strength of the struggle with the emotion.

^b^Brackets designate customized text within the questions that displayed participants’ previous choices of unwanted and valued actions.

#### User Experience Survey

In this section, we asked participants to complete a short survey, as described in experiment 1. In addition, participants assigned to the experimental group answered the following question: “Do you feel comfortable calling the avatars little monsters, or would you prefer another name?”

#### Procedure

Participants went through the same initial procedure for installing the app and providing consent as in experiment 1. In addition, this time, the app randomly assigned participants to either the control or the experimental condition.

The experiment consisted of five phases (Table 3): (1) general onboarding, (2) the 3-day preintervention phase, (3) the 7-day intervention phase, (4) the postintervention phase, and (5) the offboarding phase.

General onboarding started with a 1-minute instruction video. Participants then completed a short basic information survey (gender, age, and nationality) and the reflection module. To finish, participants in the control and experimental groups specified at what time they would prefer to receive reminder notifications to complete the evening report (between 7 PM and midnight). In addition, participants in the experimental condition specified at what time they would prefer to receive reminder notifications to complete the morning practice (between 5 AM and noon).

During the preintervention phase, all participants used a simplified version of the app, which only allowed them to complete the daily evening report. During the 7-day intervention phase, participants assigned to the experimental group were introduced to the morning practice. The introduction to the morning practice included a 1-minute video with instructions, the meta-reasoning coach, the meta-awareness coach, and a 3-minute video explaining how to use the new functionalities of the app in their everyday life. After completing the introduction, participants in the experimental condition continued with an extended version of the app that included a button to complete the morning practice, the catch function, and point rewards for practicing metacognitive skills (ie, Insight Points) in addition to the daily evening report. We asked participants assigned to the experimental group to complete the morning practice, use the catch function, and complete the evening report every day. Participants assigned to the control group continued using the simplified version of the app containing only the daily evening report. Finally, in the offboarding phase, all participants completed the poststudy user experience survey. After completing the offboarding phase, participants were directed to a screen with information to collect their payment for participation.

**Table 3:** Phases of experiment 2.^a^

Condition	General onboarding phase	Prestudy phase (3 days)	Intervention phase (7 days)	Poststudy phase (3 days)	Offboarding phase
Experimental	Reflection module	Evening survey	Morning practice Catching avatar Evening survey	Evening survey	Exit survey
Control	Reflection module	Evening survey	Evening survey	Evening survey	Exit survey

^a^The experiment consisted of five phases: (1) general onboarding, where all participants completed the reflection module; (2) the 3-day preintervention phase, where all participants completed the evening report daily; (3) the 7-day intervention phase, where participants in the control group continued to complete the evening report daily, and in addition to the evening report, participants in the experimental group completed a daily morning practice and were free to use the app to embrace their emotions during the day; (4) the postintervention phase, where all participants completed the evening report daily; and (5) the offboarding phase, where all participants filled out the exit survey and received their payment code.

#### Statistical Analysis

To test whether the outcome measures administered before and after the morning practice in experiment 2 supported the pretest-posttest results from experiment 1, we conducted a linear mixed model analysis in R (version 4.1.0; R Foundation for Statistical Computing) using the *lme4* package [27]. For each outcome measure (ie, emotion intensity, the strength of the struggle with the emotion, and the degree of endorsement of the maladaptive and adaptive beliefs), we tested whether there was a significant difference between pretest-posttest practice scores. The models contrasted the pretest-posttest scores for each outcome measure and included a random offset for each participant (*score ~ stage [1+stage|participant]*), where stage refers to either the pretest or posttest stage. Significance was calculated using the *lmerTest* package [28], which applies the Satterthwaite method to estimate *df* and generate *P* values for mixed models. We estimated the effect size using the *EMAtools* package [29], which calculates the Cohen *d* for each effect in an *lme4* object. We measured attrition if participants in the experimental and control groups differed using a chi-square test of independence. To analyze the user experience survey, we used the same procedure as in experiment 1.

## Results

### Overview

We hypothesized that the intervention would lead to significant improvements in our measures of people’s mental states after a single-session practice with the app (experiment 1). We further predicted that these improvements would also occur in participants who repeatedly interacted with the app over the course of a week (experiment 2). In addition, we hypothesized that participants would perceive the InsightApp as usable, valuable, and helpful in reducing their mental distress regarding real-life challenges. The results presented in this section support our hypotheses.

### Experiment 1

#### Single Session, Pretest-Posttest Intervention Results

**Figure 4:**
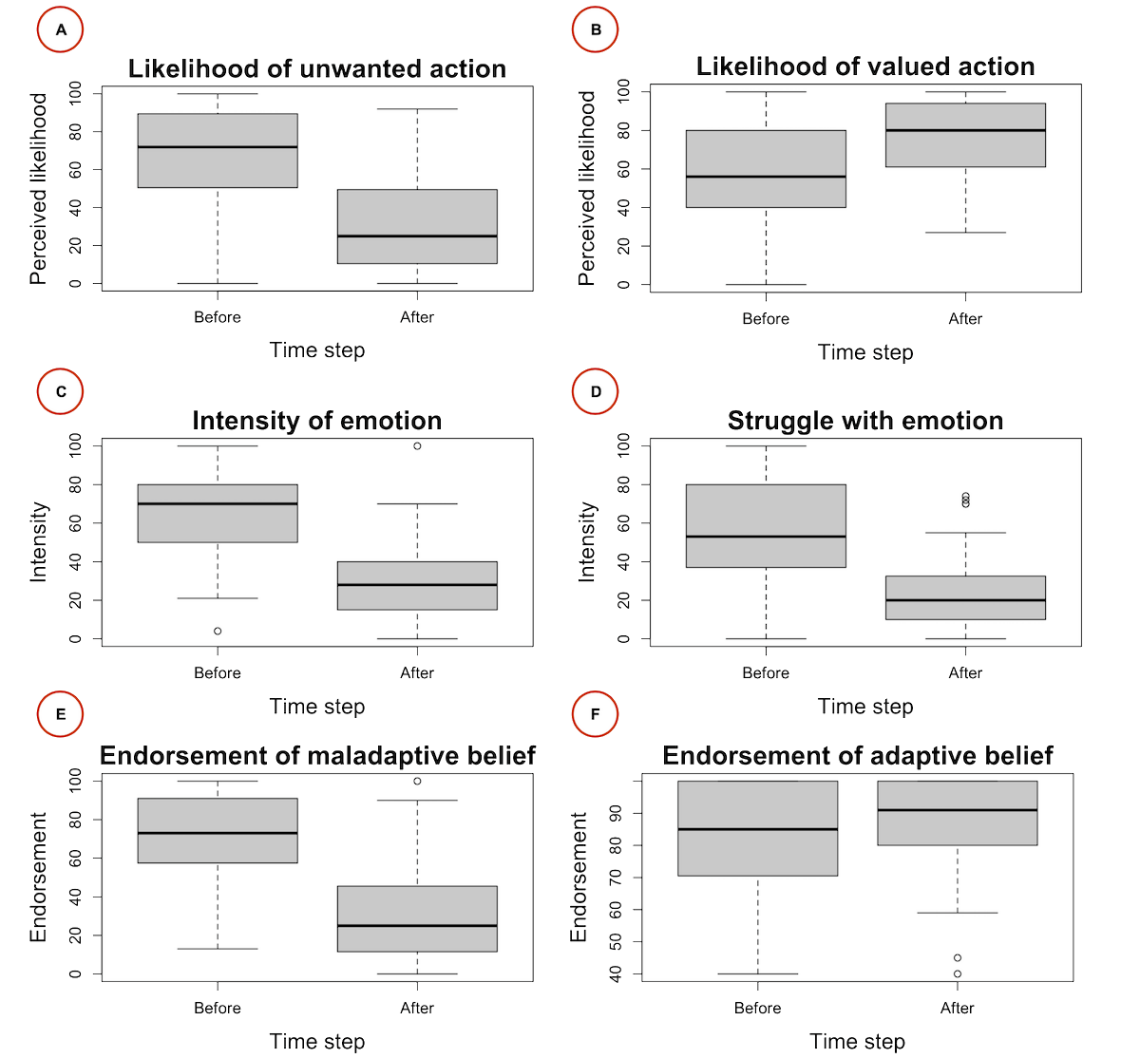
Results of experiment 1. This figure compares the outcome variables immediately before and immediately after the intervention. The scores were measured using a slider on a scale from 0 to 100. The 6 panels show scores for (A) the perceived likelihood of enacting the unwanted action, (B) the perceived likelihood of enacting the valued action, (C) the intensity of the emotion, (D) the degree of struggle with the emotion, (E) the degree of endorsement of the maladaptive belief, and (F) the degree of endorsement of the adaptive belief.

Table 4 summarizes the key descriptive statistics for each outcome measure. Figure 4 displays participants’ scores before and directly after a single practice session with the app. The results show that participants improved their scores, showing significantly decreased perceived likelihood of unwanted actions (V=88; *P*<.001; PSW effect size=0.79), increased perceived likelihood of beneficial actions (V=1549; *P*<.001; PSW effect size=0.68), decreased emotional intensity (V=1; *P*<.001; PSW effect size=0.87) and emotional struggle (V=43; *P*<.001; PSW effect size=0.83), decreased cognitive endorsement of the maladaptive belief (V=53; *P*<.001; PSW effect size=0.82), and increased cognitive endorsement of the adaptive belief (V=761; *P*<.001; PSW effect size=0.27). Thus, as predicted, by using the app, participants improved their current emotional and mental state and the perceived likelihood of acting in value-congruent ways.

Figure 4 and Table 4 show that participants’ responses are distributed over a sizable portion of the 0 to 100 ranges for each outcome measure. This suggests that slider scales are appropriate for measuring the intensity of an emotion, endorsement of a belief, and likelihood of taking a given action.

The aforementioned results suggest that our digital intervention is promising. However, as the study lacked a control condition, its results could be confounded by the study’s demand characteristics [30] and the placebo effect, among other things (please see the *Limitations* section). We see 2 possible ways to address this issue. The first is to add a control condition whereby control participants complete unrelated tasks, whereas the experimental condition interacts with the metacognitive coaches. A second improvement would be to measure and control the participants’ propensity to provide socially desirable answers [31].

**Table 4:** Descriptive statistics for participants’ pre- and postintervention scores for the intensity of their emotion, their struggle with the emotion, their perceived likelihood of performing the unwanted action and valued action, and their endorsement of their maladaptive belief and adaptive belief.

	Preintervention score				Postintervention score		
	*P* value of the Shapiro-Wilk test	Median (range)	Mean (SD)	*P* value of the Shapiro-Wilk test		Median (range)	Mean (SD)
Emotion intensity	.03	70 (4-100)	65.11 (21.37)	.006		28 (0-100)	28.57 (18.95)
Emotion struggle	.17	53 (0-100)	55.83 (26.63)	<.001		20 (0-74)	23.54 (18.58)
Unwanted action	<.001	72 (0-100)	66.37 (27.59)	<.001		25 (0-92)	31.91 (24.52)
Valued action	.08	56 (0-100)	56.54 (26.47)	<.001		80 (27-100)	76.22 (20.28)
Maladaptive belief	.004	73 (13-100)	72.24 (22.01)	<.001		25 (0-100)	31.76 (25.27)
Adaptive belief	<.001	85 (40-100)	81.52 (18.12)	<.001		91 (40-100)	87.16 (13.90)

#### User Experience Results

Multimedia Appendix 2 contains diagrams summarizing the main findings on the app’s usability and the users’ feedback for experiment 1.

When answering the following question—“What did you like or not like about the App?”—90% (57/63) of the participants commented on the positive aspects they liked about the app (panel A in Multimedia Appendix 2). The other 10% (6/63) of the participants provided feedback on aspects that could be improved. Regarding disliked features and feedback for improvement, some participants (6/63, 10%) would have preferred a more colorful or interactive interface, improved wording, and better layout of buttons, among other things. On the basis of this feedback, we see the need to improve the following features. Given that buttons are displayed in different positions depending on phone size, the next version of the app should only present buttons in the upper navigation bar. The wording of the text in the app should be reviewed and improved. In addition, before participants obtain access to the app, we should make clear that the app does not work in dark mode and should not be used on some specific iPhone models (eg, iPhone SE).

When answering regarding the degree to which they agreed with the following statement—“You developed valuable skills for your daily life” (panel B in Multimedia Appendix 2)—98% (62/63) of the participants agreed that the app taught them a valuable skill (14/63, 22% mostly agreed; 27/63, 43% agreed; and 21/63, 33% strongly agreed).

When answering the following question—“In which moments would you use the app?” (panel C in Multimedia Appendix 2)—most participants said that they would use the app to cope with their emotions as they arise during the day (32/63, 51%) and when they lack self-confidence (6/63, 10%) or motivation (4/63, 6%). Some participants (7/63, 11%) pointed out that they would like to use the app when they feel overwhelmed by emotions and need to calm down or feel better. Other participants (4/63, 6%) answered that they would like to use the app to improve their capacity for emotional awareness and self-reflection. Some participants shared that they would use the app to improve their performance (2/63, 3%) or when they feel in need of self-care (1/63, 2%), among other reasons. In total, 5% (3/63) of the participants shared that they would not use the app.

When answering the following question—“Did you find something confusing?” (panel D in Multimedia Appendix 2)—95% (60/63) of the participants responded that this was not the case, and 5% (3/63) of the participants pointed to aspects of the app or the experiment that were confusing to them and could be improved. On the basis of this feedback, we see the need to improve the breathing meditation animation by changing the arms of the avatar to a relaxed position as their facial expression changes from overwhelmed to relaxed and content.

When answering the following question—“Did you find something positively surprising?” (panel E in Multimedia Appendix 2)—92% (58/63) of the participants found some aspects of the app positively surprising. The most positively surprising aspects were creating and breathing with the animated little monster avatar (29/63, 46%) and the fact that the practice with the app was effective (19/63, 30%). Some users were also positively surprised by the fact that the app was fun and cute (7/63, 11%) and easy to use (5/63, 7%) and by the reflections and example answers (4/63, 6%). Furthermore, the average rating for the app was 4.7 out of 5 stars (panel F in Multimedia Appendix 2).

The user survey results—showing that most participants perceived the app as not confusing (60/63, 95%), positively surprising (58/63, 92%), likable (57/63, 90%), and valuable (62/63, 98%)—yielded very encouraging results regarding the feasibility, usability, and perceived effectiveness of the app. In addition, many participants (20/63, 32%) stated in open questions (when not being asked about the effectiveness of the app) that they found the app to be effective in helping them improve their current mental state when dealing with real-life challenges.

### Experiment 2

#### Overview

The results obtained in experiment 1 showed significant improvements (*P*<.001 in all cases) in the measures of people’s affective and cognitive states after a single session with the app. Furthermore, the user experience results from experiment 1 were very positive concerning the app’s usability, value, and helpfulness in improving their mental distress regarding real-life challenges. In experiment 2, we tested whether these results could be replicated in a more naturalistic setting where participants repeatedly interacted with the app over the course of a week. Another major goal of this experiment was to assess the feasibility of conducting a randomized controlled trial using the current version of the app. To determine this, we measured attrition and compared the attrition rates between the experimental condition and a control condition.

#### Attrition

The attrition rate was significantly higher in the experimental condition (41/100, 41%) than in the control condition (17/100, 17%; *χ*^2^_1_=12.8, *P*<.001). This asymmetric attrition suggests that the experimental design delivered by the first prototype of the InsightApp was suboptimal for longitudinal studies. We attribute the asymmetric dropout rates to 2 main causes. The first is that the experimental condition required considerably more time and effort than the control condition. Concretely, the app required the experimental group to complete an introduction to the morning practice at the end of the prestudy period, complete a daily morning practice, and actively use the metacognitive coaches to embrace anxiety during the intervention period in addition to the daily evening report. In contrast, the control group only had to consistently complete the daily evening report. The second related cause is that the list of requirements that the experimental group had to fulfill to avoid being excluded was much longer than the corresponding list for the control group.

To address these issues, future versions of the InsightApp will provide the control group with additional, unrelated tasks that take approximately as much time and effort as those of the experimental group. In place of the experimental group’s meta-reasoning task, the control group will be asked a series of unrelated questions about their preferences. In place of the morning practice with the meta-awareness coach, the revised control condition will present participants with unrelated cognitive tasks (eg, a Stroop task or a spatial memory task) from the ResearchKit (Apple Inc) open-source library [32].

#### Multiple Sessions, Pretest-Posttest Intervention Results

In experiment 1, we found that a single practice training session with the app can significantly improve participants’ mental state. In experiment 2, we studied whether those results were replicated by the within-participant pretest-posttest measures of their daily morning practice session. As expected, the results of the longitudinal experiment support the results obtained in experiment 1. For each of the dependent variables, there was a significant improvement from before the training session to immediately after the training session. After the morning practice, participants showed significantly decreased scores for emotion intensity (*t*_316.7_=−5.959; *P*<.001; Cohen *d*=−0.67), emotional struggle (*t*_236.4_=−5.048; *P*<.001; Cohen *d*=−0.66), and cognitive endorsement of the discouraging belief (*t*_610.6_=−7.385; *P*<.001; Cohen *d*=−0.60). In addition, participants showed significantly increased scores for cognitive endorsement of the encouraging belief (*t*_656.1_=3.642; *P*<.001; Cohen *d*=0.28). Table 5 summarizes the effects of our intervention on each outcome measure for experiments 1 and 2.

**Table 5:** Difference in pretest-posttest measures directly after completing a session with the app.^a^

Outcome measure	Experiment 1—single sessions				Experiment 2—repeated measures		
	Effect size	Magnitude	*P* value	Effect size		Magnitude	*P* value
Intensity of the emotion	−0.87	Large	<.001	−0.67		Large	<.001
Strength of the struggle	−0.83	Large	<.001	−0.66		Large	<.001
Strength of maladaptive belief	−0.82	Large	<.001	−0.60		Large	<.001
Strength of adaptive belief	0.27	Small	<.001	0.28		Small	<.001
Likelihood of unwanted action	−0.79	Large	<.001	—^b^		—	—
Likelihood of valued action	0.68	Large	<.001	—		—	—

^a^The common interpretation for effect size values for paired Wilcoxon tests in published literature is small effect (0.10 to <0.3), moderate effect (0.30 to <0.5), and large effect (≥0.5) [33].

^b^In experiment 2, pretest-posttest changes in participants likelihood of unwanted and valued action were not measured.

#### User Experience Results

Multimedia Appendix 3 contains diagrams summarizing the main findings on the app’s usability and the users’ feedback for experiment 2.

When answering the following question—“What did you like or not like about the App?”—56% (33/59) of the participants only commented on aspects they liked about the app. A total of 44% (26/59) of the participants shared both positive aspects they liked and aspects they disliked or could be improved. The most appreciated aspects are summarized in panel A in Multimedia Appendix 3. For testing purposes and to remove the variance between participants, we developed a minimal version of the app with limited functionalities (eg, participants could work with only 1 emotion). Some feedback pointed to removing these limitations or provided ideas for new functionalities. In this section, we only address the negative feedback that is useful for improving the current version of the app to conduct longitudinal studies. From this feedback, we learned that it is important to improve how we deliver information about the study timeline to participants. This could be accomplished by sending them daily messages with information about their daily goals, their progress, and the next steps. It will also be important to insert a trigger warning in the instructions highlighting the fact that the app will ask them to reflect on a difficult situation and get in touch with difficult emotions. Regarding the evening report, it would be useful to add a new question asking if there was another unrelated situation that caused anxiety that day and use it as a covariate. Regarding the morning practice, we see the need to add an option to allow people to keep breathing after finishing the morning practice and choose how many breaths to take. Regarding the “catch function,” an important improvement would be to add random periodic reminders during the day for participants to be aware of their mental state and use the app to breathe when feeling overwhelmed by the troubling emotion. The study also identified bugs that occur only in specific cases, such as the absence of notifications or misconfigured calendars, and in-app text that needs to be reworded. We will address all these issues in the next version of the InsightApp.

When answering regarding the degree to which they agreed with the following statement—“You developed valuable skills for your daily life.” (panel B in Multimedia Appendix 3)—86% (51/59) of the participants agreed (17/59, 29% mostly agreed; 30/59, 51% agreed; and 4/59, 7% strongly agreed) that the app taught them a valuable skill for daily life.

When answering the following question—“In which moments would you use the app?” (panel C in Multimedia Appendix 3)—most participants (33/59, 56%) answered that they would use the app to cope with their emotions as needed during the day, and a subset of participants (9/59, 15%) answered that they would like to use the app to start or end the day. Some participants (5/59, 8%) would use the app when feeling overwhelmed by emotions and needing to calm down or feel better. Other participants pointed out that they would like to use the app to actively improve their capacity for self-reflection and emotional awareness (3/59, 5%), when lacking self-confidence (1/59, 2%) or motivation (2/59, 3%), and for changing habits (1/59, 2%), among other reasons. In total, 6% (4/59) of the participants shared that they would not use the app.

When answering the following question—“Did you find something confusing?”—83% (49/59) of the participants responded *no* (panel D in Multimedia Appendix 3). A total of 17% (10/59) of the participants pointed to aspects of the app or the experiment that were confusing for them and that could be improved. A total of 8% (5/59) of the participants commented that the changes between different phases of the experiment were confusing and that it was not clear when each stage would take place. In total, 5% (3/59) of the participants found the wording of some questions confusing. A total of 3% 2/59) of the participants would have liked to receive instructions regarding the process of creating the avatar in advance. In total, 2% (1/59) of the participants were confused about how to answer the evening report as they experienced anxiety that was unrelated to the presence of the prespecified stressful situation. Another participant was confused by the weekly calendar, which was misconfigured on their phone. In addition, 2% (1/59) of the participants commented that the introduction videos were helpful for avoiding confusion.

When answering the following question—“Did you find something positively surprising?” (panel E in Multimedia Appendix 3)—73% (43/59) of the participants found some aspects of the app positively surprising. Similar to the results obtained in experiment 1, most users were positively surprised by the fact that the practice with the app was effective for them (23/59, 38%) and by the process of creating or breathing with the animated little monster avatar (14/59, 24%). Some users were also surprised by the fact that the app was fun (3/59, 5%) and easy to use (3/59, 5%), that it improved their performance during the day (3/59, 5%), and that breathing when feeling a strong emotion became habitual for them (1/59, 2%). Other participants were positively surprised by the reflections (2/59, 3%) and the fact that they gained insights into their mental world (3/59, 5%), among other things.

The average rating for the app was 4 out of 5 stars (panel F in Multimedia Appendix 3). In addition, we asked the experimental group to share their opinions about calling the avatars “little monsters.” In total, 81% (48/59) of the participants liked the name, 7% (4/59) were indifferent, and 12% (7/59) did not like the name or suggested changing it. Some suggestions for new names were “Little Helpers,” “Demons,” and “Widgets.”

The user experience survey for the 2-week study—showing that most participants perceived the app as not confusing (49/59, 83%), positively surprising (43/59 73%), likable (51/59, 86%), and valuable (51/59, 86%)—corroborates the positive results obtained in experiment 1 regarding usability with perceived effectiveness and the usefulness of the app. In addition, the survey results from experiment 2 provided feedback on disliked features and how they could be improved.

## Discussion

### Principal Findings

We conducted 2 formative studies to test the feasibility, usability, and potential effectiveness of the InsightApp in helping people leverage metacognitive strategies to embrace challenging situations and cope with emotional distress in real-life settings. As hypothesized, the results from experiment 1 indicated that a single session with the app helps people improve their emotional state, the degree to which they believe adaptive versus maladaptive thoughts, and their perceived likelihood of acting in value-congruent versus unwanted ways. Experiment 2 indicated that these benefits are almost equally large when people repeatedly use the InsightApp over the course of a week. Thereby, we replicated the findings of experiment 1 under more naturalistic conditions. Furthermore, the results from the user experience surveys supported the hypothesis that participants perceived using the InsightApp as a feasible, valuable, and helpful way to cope with stressful situations in their everyday lives. The exit surveys also provided valuable feedback on how to improve the experiment’s instructions and several of the app’s features.

Regarding the feasibility of using the InsightApp as a research tool, the results from experiments 1 and 2 provided valuable information on which features work (eg, example answers and rating degrees of affect, belief, and behavior using sliders) and which ones do not work (eg, the design of the control condition). The preliminary studies presented in this paper are an important step toward the design and evaluation of an app that leverages techniques from different psychotherapies for training metacognitive skills for people to cope, in a healthy way, with the difficult emotions they experience in stressful situations.

### Limitations

The formative studies presented in this paper have both strengths and limitations. Among the strengths of the preliminary studies are the relatively large sample sizes and the replication of results across the experiments. In addition, we sampled from a relatively large and varied population, which is more diverse and representative than the undergraduates who typically participate in psychological experiments. Furthermore, participants interacted with a high-fidelity prototype of the app. Therefore, the results are highly informative of how users will respond to the final version of the InsightApp.

Among the limitations of the studies are the lack of a control condition in experiment 1 and the asymmetric attrition in experiment 2, which prevented us from comparing the results of the experimental condition with those of the control condition. The use of single-item measures is another possible limitation, comprising several outcome measures collected by the app. There is a concern that the reliability of single-item measures may be objectionably low [34]. To measure the reliability of single-item measures, we evaluated the test-retest reliability of our single-item measures in a follow-up study (Multimedia Appendix 1). Other potential limitations of our studies are the confounding effects. We identified 3 main potential confounding factors. The first confounding factor is participants’ expectations that the intervention is effective and will help them improve their mental state (ie, the placebo effect). The second confounding factor is participants’ potential implicit desire to comply with the goals of the experiment (ie, demand characteristics). Finally, the third potential confounding factor, as reported by Tamir et al [35], is that the preintervention stage might activate in participants the goal of improving their emotion, which can lead to the activation of emotion regulation mechanisms by itself. Another limitation of the studies is that we did not measure whether participants actually performed the mindful breathing exercise. We only measured the effect of participants being told to perform the exercise, which is generally lower than the effect of actually performing it. Moreover, our participants were not fully representative of the app’s potential users. Our participants were paid to complete the study. Therefore, they may have been more willing to invest effort and be patient with our app than real users. In addition, real users who will seek out our app on the App Store will likely be experiencing higher amounts of mental distress. Furthermore, to use the app, it is key for users to understand the reflections and instructions. Our sample from Prolific likely had more experience answering psychological questions than the general population.

### Comparison With Prior Work

In this section, we compare the effect sizes obtained in our studies with those commonly obtained by EMIs for improving mental health, mental health apps, and mindfulness interventions. It is important to keep in mind that most previous studies have evaluated the pretest-posttest effects of different interventions in terms of the improvements achieved over days, weeks, or months. In contrast, in experiments 1 and 2, we evaluated the active components of the intervention in terms of immediate improvement after completing a single session with the app that lasted between 5 and 30 minutes. On the one hand, we measured the effect of a much lower dose of the intervention. In contrast, treatment effects tend to decay over time. Furthermore, the formative results obtained regarding our app did not include a control condition, which is the case in most studies regarding the effectiveness of interventions. Therefore, given the differences in timescales and experimental design, it is not clear whether and, if so, how our effect sizes can be compared with effect sizes from other studies. Nevertheless, we compared our results with those of similar previous interventions. Future work should measure the effectiveness of the InsightApp in the same way in which it is measured by existing interventions. This would make it possible to select between alternative interventions based on comparable measures of efficacy.

The InsightApp average pretest-posttest scores for participant improvements in affect—measured through a decrease in the intensity of perceived negative emotion and the degree to which they struggled with it—revealed large effects when measured once in experiment 1 (average Cohen *d*=−0.85) or multiple times in experiment 2 (average Cohen *d*=−0.67). These effects are noticeably larger than the average effects reported in previous studies evaluating the effectiveness of EMIs in improving mental health [10], mental health apps [35], and mindfulness interventions [7]. However, these comparisons should be taken with a grain of salt as we measured the efficacy of our app within the experimental group, whereas the meta-analytic effect sizes are mostly based on studies comparing the experimental condition with a control condition. Moreover, we measured effect sizes using the Cohen *d*, whereas the meta-analyses with which we are comparing measured effect sizes using the Hedges *g*. However, Hedges *g* effect sizes are comparable with Cohen *d* effect sizes given samples of >20 participants. In the following paragraph, we summarize the findings of the most relevant prior work in more detail.

According to a meta-analysis of 33 EMI studies [10], most of the interventions focused on clinical samples, and half of the studies’ EMIs were used with the support of mental health professionals. On average, the studies lasted 7.5 weeks and delivered from 4 to 420 training episodes. Most of the interventions provided active training, which included exercises (76%). Similarly, the InsightApp delivered an active intervention offering exercise sessions. Our intervention presented an average of 17 training episodes distributed over a period of 1 week (experiment 2). Similar to our formative experiments, 17 of the studies included in the meta-analysis used a pretest-posttest design. The within-subject analyses showed a small to medium effect for EMIs that were not supported by mental health professionals (*g*=0.45, 95% CI 0.22-0.69). The average effect was small to medium for stress (*g*=0.40, 95% CI 0.23-0.57) and anxiety (*g*=0.47, 95% CI 0.32-0.63). The meta-analysis by Firth et al [36] of randomized clinical trials testing the efficacy of mental health apps for clinical and high subclinical levels of anxiety or depression found small effects (*g*=0.33, 95% CI 0.17-0.48; *P*<.01). The average effect sizes found for mindfulness interventions targeting the general population were small for perceived stress (*g*=0.46, 95% CI 0.24-0.68) and anxiety (*g*=0.28, 95% CI 0.16-0.40) [7].

In our second experiment, the adherence scores were asymmetric between the experimental (60%) and the control (82%) conditions. Despite the asymmetric dropout between conditions, the adherence score for the experimental condition was comparable with the adherence scores for mobile mental health apps targeting the clinical population (ie, 56.6%) and mindfulness apps targeting the general public (ie, 59%), as reported by Jakob et al [37]. The review by Jakob et al [37] covered 20 mental health apps with a median intervention length of 56 days and 9 mindfulness apps with a median intervention length of 42 days. It is important to consider that there is a series of factors influencing the adherence rates of experiments 1 and 2, which complicates the comparison with other studies. First, participants in both studies were paid and motivated with bonuses to complete the studies. Second, the samples collected for both studies were not clinical. In contrast, the exclusion criteria for experiment 2 were strict in the sense that they required a minimum of 70% compliance.

### Conclusions

In this paper, we presented 2 formative studies to test the feasibility, usability, and potential effectiveness of the InsightApp in helping users leverage metacognitive strategies to embrace challenging situations and cope with emotional distress in real-life settings. The encouraging preliminary results indicate that it is worthwhile to continue developing the InsightApp and evaluate it in a randomized controlled trial. Furthermore, the results from the user experience surveys supported the hypothesis that participants perceived using the InsightApp as a feasible, valuable, and helpful way to cope with stressful situations in their everyday lives. The exit surveys also provided valuable feedback on how to improve the experiment’s instructions and the features of the app.

## Appendix

Multimedia Appendix 1 Reliability of single-item measures.

Multimedia Appendix 2 The InsightApp’s usability ratings and user feedback for experiment 1. Panel (A) shows the number of participants who mentioned liking different aspects of the app. Panel (B) shows the degree to which participants agreed that the app taught them a valuable skill. Panel (C) shows participants’ preferences regarding when to use the app. Panel (D) shows the number of participants who found the app confusing. Panel (E) shows the number of participants who were positively surprised by the app. Panel (F) shows participants’ ratings of the app on a scale from 1 to 5 stars.

Multimedia Appendix 3 The InsightApp’s usability ratings and user feedback for experiment 2. Panel (A) shows the number of participants who mentioned liking different aspects of the app. Panel (B) shows the degree to which participants agreed that the app taught them a useful skill. Panel (C) shows participants’ preferences regarding when to use the app. Panel (D) shows the number of participants who found the app confusing. Panel (E) shows the number of participants who were positively surprised by the app. Panel (F) shows participants’ ratings of the app on a scale from 1 to 5 stars.

## Abbreviations

ACT: acceptance and commitment therapy
CBT: cognitive behavioral therapy
EMI: ecological momentary intervention
MBI: mindfulness-based intervention
PSW: paired-sample Wilcoxon test

## Data Availability

The data sets generated and analyzed during this study are available in Open Science Framework storage, *Metacognitive coaches formative studies* repository [38].
