# Supplementary material for: A Gamified Mobile App That Helps People Develop the Metacognitive Skills to Cope With Stressful Situations and Difficult Emotions: Formative Assessment of the InsightApp
Source: JMIR Form Res. 2023 Jun 16;7:e44429. doi: 10.2196/44429 (PMC10337330; doi:10.2196/44429)
Supplement: Multimedia Appendix 1 [file formative_v7i1e44429_app1.docx]

## Multimedia Appendix 1

### Experiment 3: Reliability of single item measures

In Experiment 1 and 2, we used a pre-post design to test how effective the app is at helping people improve their current mental states. In Experiment 3, we measured the test-retest reliability of those single-item measures. Experiment 3 aimed to evaluate how adequate the single-item slider measures used in the app are as outcome variables.

#### Participants

The sample consisted of 170 participants (completion rate: 80%) from a population of approximately 42 thousand, English-speaking participants on Prolific. The study was divided into two sessions, the test survey session and the retest survey session. Of the 170 participants who started the experiment, 137 completed both sessions. Seven of these were excluded from the analyses since they reported having a technical problem with the survey, leaving a final sample of 130 participants. From those, 223 shared their demographic data (average age: 38 years, range: 19–75 years, female n=105, 47%). The sample included participants receding in the USA who identified as white (n = 141, 63%), Asian (n = 45, 20%), black (n = 16, 7%), mixed (n = 5, 2%), or other (n = 16, 7%).

Each participant was paid 9 £/hr. The mean completion time was 4 minutes for the first session and 3 minutes for the retest session.

#### Outcome measures

Table 6, summarizes the outcome measures collected by the survey. The questions asked in the survey were identical to that of the single-item measures in the app (i.e., the wording and the system of sliders for rating). The process of selecting emotions and beliefs closely resembled the process implemented in the app. There were, however, three differences between the app and the surveys. First, the surveys were taken online and not necessarily using a mobile device. Second, participants had to select an option from the example answers and could not write free-text. Third, in order to avoid delivering the entire meta-reasoning intervention, we removed the word “thoughts” out of the questions measuring the endorsement of beliefs and replaced them with the actual belief participants had previously chosen. In addition to the outcome measures collected in the app, the survey included a list of unrelated questions regarding participants preferences. The data sets from the completed surveys can be found in the Supplementary Material.

Table 6. Single-item outcome measures and unrelated questions used in Experiment 3^a,b^

| Outcome variable | Question | Scale |
| --- | --- | --- |
| Intensity of the emotion | Which of the following emotions are you experiencing the most the past week?  **How intense does [emotion] feel right now?** | Multiple choice  **0-100%** |
| Strength of the struggle | **How strong is the struggle with [emotion] right now?** | **0-100%** |
| Likelihood of unwanted action | In which UNWANTED ways do you tend to act when you feel [negative_emotion]?  **How likely are you to [unwanted action] in your current emotional state?** | Multiple choice  **0-100%** |
| Likelihood of  valued action | In which VALUED ways you would like to act?  **How likely are you to [valued action] in your current emotional state?** | Multiple choice  **0-100%** |
| Strength of Maladaptive belief | What do you tend to think when you feel [negative_emotion]? (choose closest match)  **How much do you believe [maladaptive_belief] right now?** | Multiple choice  **0-100%** |
| Strength of adaptive belief | What do you tend to think to feel better? (choose closest match)  **How much do you believe [adaptive_belief] right now?** | Multiple choice  **0-100%** |
| Unrelated questions | Which of the following skills would you prefer to have?  How much do you value {preferred_skills} skills?  How hungry do you tend to be during the day?  How much do you feel like walking outside on a normal day?  Who do you prefer to spend time with?  How much do you feel like spending time with {preferred_company} normally?  How much do you feel like eating sweet foods normally?  How much do you feel like eating salty foods normally?  At what time of the day are you usually at your best?  How much do you feel like socializing in general? | Multiple choice  0-100%  0-100%  0-100%  Multiple choice  0-100%  0-100%  0-100%  Multiple choice  0-100% |

^a^The single-item outcome measures are highlighted in bold. The surveys asked participants to use a slider (0 to 100%) to rate (1) the intensity of the negative emotion they are feeling (2) the degree to which they are struggling with the emotion, (3) the current perceived likelihood of acting in unwanted ways, (4) the current perceived likelihood of acting in a value-congruent way, (5) their endorsement of the maladaptive belief, and (6) their endorsement of the adaptive belief.

^b^Brackets (“[ ]”) designate customized text within questions, which display participants' previous choices of emotion, beliefs, and tendencies to action.

#### Procedure

Participants completed the survey twice within a 1.5–6 hours window. The average delay between the test and the retest was 2:26h.

#### Statistical analysis and results

To estimate the reliability of single-item measures, we calculated the Pearson correlation between the test and retest scores. All measured correlations were at least 0.6. Despite the large sample, none of the reliabilities were significantly different than 0.7, which is considered acceptable reliability (all p ≥ 0.366).

Table 7. Single-item outcome measures test-retest result.^a-d^

| Single item measure | Reliability | 95% CI | P-value | Interpretation |
| --- | --- | --- | --- | --- |
| Intensity of the emotion | r(128) =0.69 | [0.60, 1.00] | P = 0.607 | Not significantly different from acceptable reliability. |
| Strength of the struggle | r(128) =0.72 | [0.62, 0.79] | P = 0.366 | Not significantly different from acceptable reliability. |
| Likelihood of unwanted action | r(128) =0.6 | [0.48, 0.70] | P = 0.97 | Not significantly different from acceptable reliability. |
| Likelihood of valued action | r(128) =0.66 | [0.54, 0.74] | P = 0.82 | Not significantly different from acceptable reliability. |
| Strength of Maladaptive belief | r(128) =0.6 | [0.48, 0.69] | P = 0.71 | Not significantly different from acceptable |
| Strength of adaptive belief | r(128) =0.67 | [0.57, 0.76] | P = 0.97 | Not significantly different from acceptable reliability. |

^a^ All *P*<0.001  for H₀:  r ≠ 0

^b^  95% Confidence Interval

^c^Significance level (α=0.05) for H₀:  r <0.7

^d^All single-item measures reliability are not significantly different from 0.7, what is considered  acceptable reliability.
