# Supplementary figures and images for: A Gamified Mobile App That Helps People Develop the Metacognitive Skills to Cope With Stressful Situations and Difficult Emotions: Formative Assessment of the InsightApp
Source: JMIR Form Res. 2023 Jun 16;7:e44429. doi: 10.2196/44429 (PMC10337330; doi:10.2196/44429)

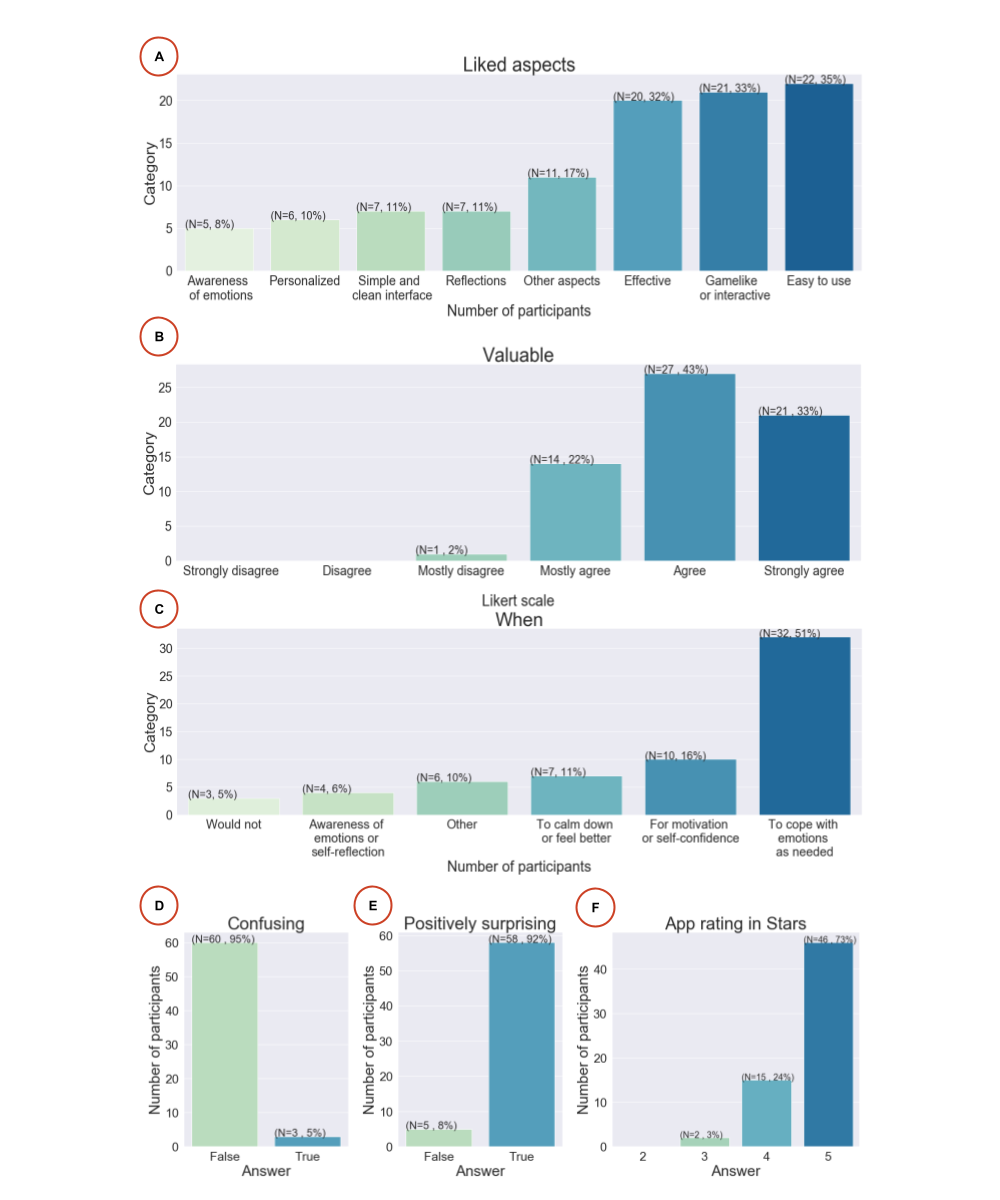

Supplement: Multimedia Appendix 2 [file formative_v7i1e44429_app2.png]

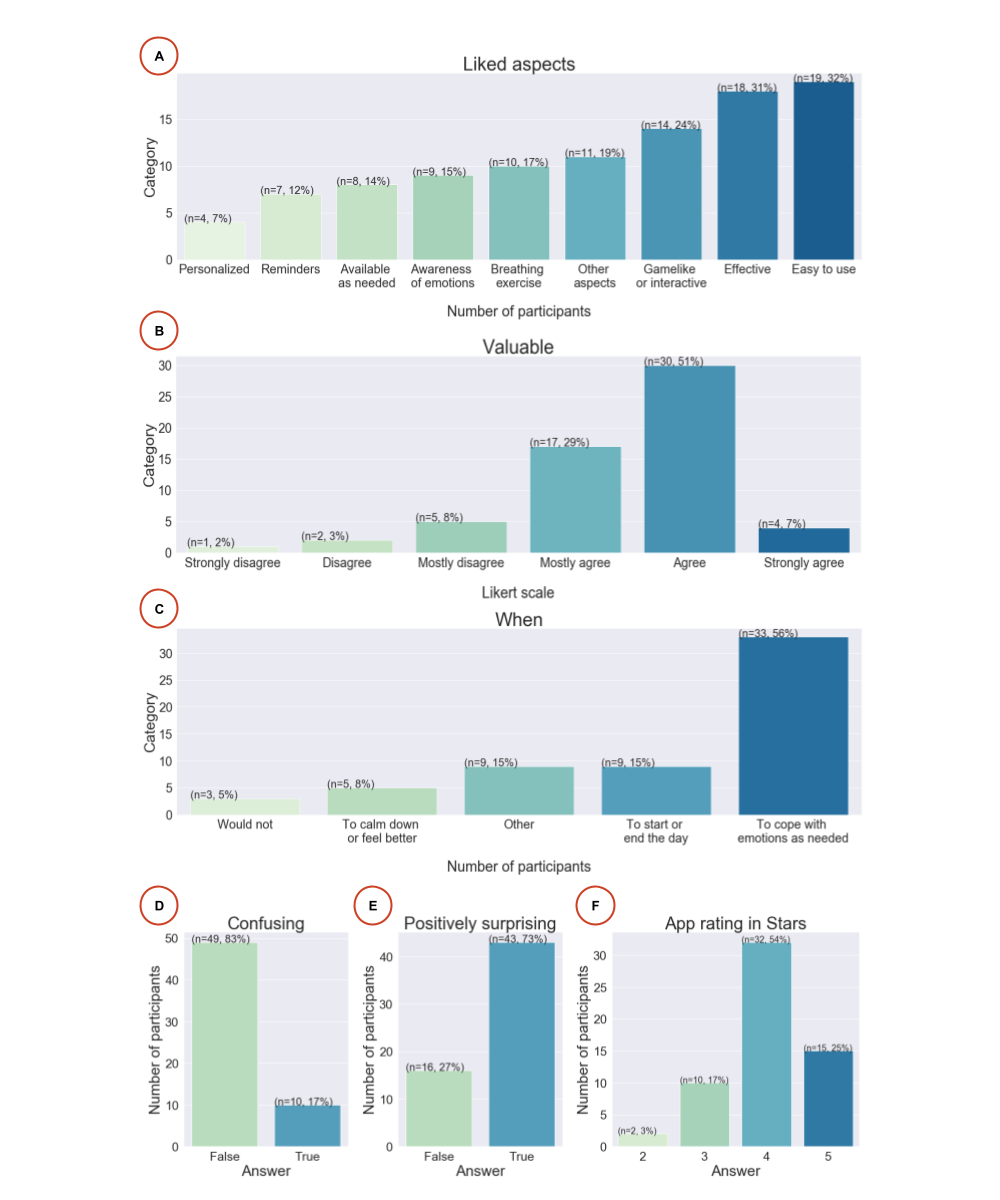

Supplement: Multimedia Appendix 3 [file formative_v7i1e44429_app3.png]
